# Pedestrian Detection Based on Adaptive Selection of Visible Light or Far-Infrared Light Camera Image by Fuzzy Inference System and Convolutional Neural Network-Based Verification

**DOI:** 10.3390/s17071598

**Published:** 2017-07-08

**Authors:** Jin Kyu Kang, Hyung Gil Hong, Kang Ryoung Park

**Affiliations:** Division of Electronics and Electrical Engineering, Dongguk University, 30 Pildong-ro 1-gil, Jung-gu, Seoul 100-715, Korea; kangjinkyu@dgu.edu (J.K.K.); hell@dongguk.edu (H.G.H.)

**Keywords:** pedestrian detection, visible light and FIR cameras, fuzzy inference system, adaptive selection, convolutional neural network

## Abstract

A number of studies have been conducted to enhance the pedestrian detection accuracy of intelligent surveillance systems. However, detecting pedestrians under outdoor conditions is a challenging problem due to the varying lighting, shadows, and occlusions. In recent times, a growing number of studies have been performed on visible light camera-based pedestrian detection systems using a convolutional neural network (CNN) in order to make the pedestrian detection process more resilient to such conditions. However, visible light cameras still cannot detect pedestrians during nighttime, and are easily affected by shadows and lighting. There are many studies on CNN-based pedestrian detection through the use of far-infrared (FIR) light cameras (i.e., thermal cameras) to address such difficulties. However, when the solar radiation increases and the background temperature reaches the same level as the body temperature, it remains difficult for the FIR light camera to detect pedestrians due to the insignificant difference between the pedestrian and non-pedestrian features within the images. Researchers have been trying to solve this issue by inputting both the visible light and the FIR camera images into the CNN as the input. This, however, takes a longer time to process, and makes the system structure more complex as the CNN needs to process both camera images. This research adaptively selects a more appropriate candidate between two pedestrian images from visible light and FIR cameras based on a fuzzy inference system (FIS), and the selected candidate is verified with a CNN. Three types of databases were tested, taking into account various environmental factors using visible light and FIR cameras. The results showed that the proposed method performs better than the previously reported methods.

## 1. Introduction

A number of studies are currently being conducted with a view to increasing the accuracy of the pedestrian detection schemes as intelligent surveillance systems are being advanced. In the past, visible light cameras were widely used [1,2,3,4,5,6,7], however, these cameras are quite vulnerable to factors such as varying shadows and lighting, and cannot accurately detect pedestrians during nighttime. To address such constraints, numerous studies on pedestrian detection systems using far-infrared (FIR) light cameras (thermal cameras) are being conducted [7,8,9,10]. However, pedestrian detection remains a difficult challenge as the differences between the pedestrian and the non-pedestrian areas decrease as the solar radiation causes the air temperature to reach the body temperature level. In order to address such issues, researchers have been exploring methods to use both visible light and FIR camera images. This includes a method of selecting the visible-light and thermal-infrared images under the dynamic environments as presented in [11], and a method of detecting the pedestrians by combining these two images [12,13,14]. 

However, these methods may increase the processing time and computational complexity as they have to take into account both visible light and FIR camera images, and process the convolutional neural network (CNN) twice [13]. In order to overcome these limitations, our research suggests a method that is able to detect the pedestrians under varying conditions. The proposed method is more reliable than a single camera-based method, reduces the complexity of the algorithm, and requires less processing time compared to the methods using both visible light and FIR camera images. This is because our method adaptively selects one candidate between two pedestrian candidates derived from visible light and FIR camera images based on a fuzzy inference system (FIS). To enhance the detection accuracy and processing speed, only the selected one candidate is verified by the CNN. 

The scenario where our system can be applied is the pedestrian detection by intelligent surveillance cameras in outdoor environments. Therefore, all the experimental datasets were collected considering this environment as shown in Section 4.1. The detected position of pedestrians by our method at various times and in different environments can be used as basic information for face recognition, behavior recognition, and abnormal pedestrian case detection, which are necessary for crime and terror prevention, and the detection of emergency situations where a person suddenly falls down on the street and does not move. The following Section 2 looks extensively into various pedestrian detection scheme studies.

## 2. Related Works

The pedestrian detection studies that are available to date can be divided into two groups: (a) single camera-based methods (infrared or visible-light cameras) [6,10,15,16,17,18,19,20,21,22], and (b) multiple camera-based methods [11,12,13,22,23,24]. The former group includes the following methods: (i) adaptive boosting (AdaBoost) cascade-based method, which is widely used as the representative facial detection scheme [25,26], (ii) histogram of oriented gradient-support vector machine (HOG-SVM) method [18], (iii) integral HOG [19] method, whose processing speed was reported to be significantly faster than the existing HOG, (iv) neural network-based method using the receptive field approach [27] for pedestrian detection [20], and (v) methods based on background generation with FIR cameras [21]. However, these single camera-based methods have a common constraint that their detection performance degrades when their surroundings vary. For instance, a visible light camera-based method barely detects the pedestrians during dark nights, and is affected by varying shadows and lighting. Similarly, an FIR camera-based method cannot detect the pedestrians when bright sunshine increases the ground temperature up to the body temperature level.

To address these issues, studies on CNN-based pedestrian detection are being conducted. John et al. used an FIR camera to study how to detect pedestrians based on adaptively fuzzy c-means clustering and CNN [10]. Considering the daytime and the nighttime conditions, the researchers suggested a more resilient algorithm. This work, however, did not include experiments under conditions where the aforementioned background air temperature was similar to that of the pedestrians. In the study of the pedestrian detection with a CNN [6], the authors showed that the large margin CNN method outperformed the SVM method in pedestrian detection using a visible light camera. However, this study did not include experiments on images under varying environmental factors, such as varying lighting and shadows. Such CNN-based pedestrian detection methods showed better performance compared to the previously studied methods while they still failed to overcome the limitations associated with the varying environmental conditions, such as, varying lighting and shadows, and the cases where the background had the same temperature as the pedestrians. 

To address the above limitations, multiple camera-based detection methods were also being studied. In a study involving multi-cue pedestrian detection and moving vehicle tracking [23], the authors proposed a stereo visible light camera-based pedestrian detection method that employs shape and texture information. Bertozzi et al. suggested an HOG-SVM-based pedestrian detection system based on tetra-vision using visible light and FIR camera images [24]. It used a vehicle’s headlights and a combination of visible light and FIR camera images for pedestrian detection purposes. This method was validated for nighttime conditions, which took a longer time to process. Another study on a multi-spectral pedestrian detection method [22] using both visible light and near-infrared (NIR) light camera images was conducted using HOG-SVM. In contrast, Serrano-Cuerda et al. conducted a study on pedestrian detection systems under a more diverse environmental setting than the aforementioned studies [11]. As the detection performance of the cameras appeared vulnerable to the weather and environmental conditions, the study used confidence measures (based on the mean lighting and standard deviation information) to select the more appropriate images from visible light and FIR camera images. 

Lee et al. combined visible-light and FIR camera-produced pedestrian data based on difference images, and suggested a method for detecting the pedestrians [12]. However, there exists a doubt that the cameras discussed in [11] and in [12] may have lower performance as no final verification was provided in those publications. In addition, Wagner et al. suggested two methods in their study [13]. The first method was an early fusion CNN method, which converged both the visible light and FIR images, that were fed to the CNN as inputs. The second method, called the late fusion CNN-based method, employed training of the pedestrian and the background domains (each from visible light and FIR images), and converging the features collected from the fully connected layers. Among the two, the latter showed a better performance. However, this method may increase the processing time and computational complexity as it has to take into account of visible light and FIR camera images, and process the CNN twice. 

In order to overcome these limitations, this paper suggests a method that is able to detect the pedestrians under varying conditions. It is novel in the following three ways compared to the previously published works:

- The proposed method is more reliable than a single camera-based method, reduces the complexity of the algorithm, and requires less processing time compared to the methods using both visible light and FIR camera images. This is because our method adaptively selects one candidate between two pedestrian candidates derived from visible light and FIR camera images based on a fuzzy inference system (FIS).
- The two input features of FIS vary owing to the fact that the input candidate images are of the following types: pedestrian or non-pedestrian (background). Therefore, to remove such uncertainties, this study applies Gaussian fitting to the distribution of the gradient-based features of the input candidate images, and adds weights (resulting from such a fitted Gaussian distribution) to the FIS output. By doing so, it enables a more accurate and adaptive selection process for the FIS regardless whether the images were pedestrian type or non-pedestrian type.
- It increases the accuracy of the pedestrian detection process by verifying the FIS-selected pedestrian candidate through the CNN. In addition, we have opened our database and trained CNN model to other researchers in order to compare the performances.

Table 1 shows a comparison of the proposed and the previously researched pedestrian detection methods, including their respective advantages and disadvantages. The remainder of this paper consists of the following sections: Section 3 presents the details of the concepts behind the proposed system. The experimental results and various performance comparisons (among the existing methods) are presented in Section 4. Finally, Section 5 provides our conclusions.

## 3. Proposed Method

### 3.1. Overall Procedure of the Proposed System

**Figure 1:**
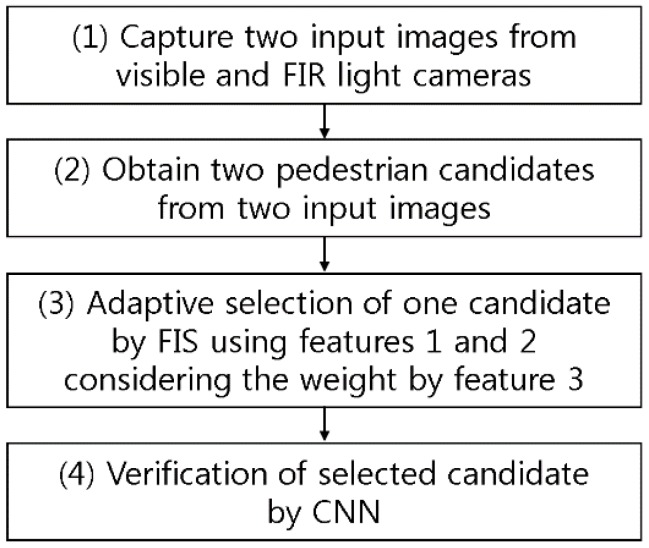
Overall procedure of the proposed system.

**Figure 2:**
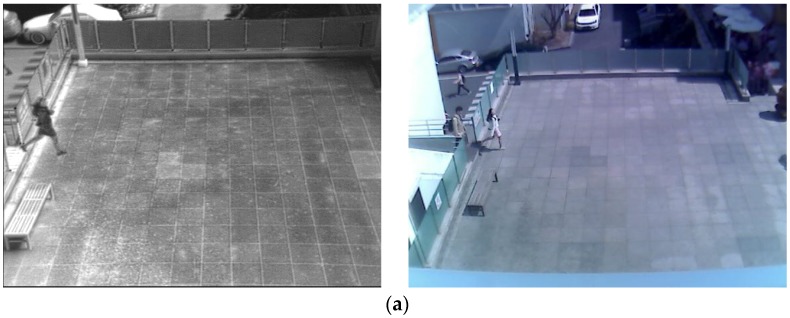

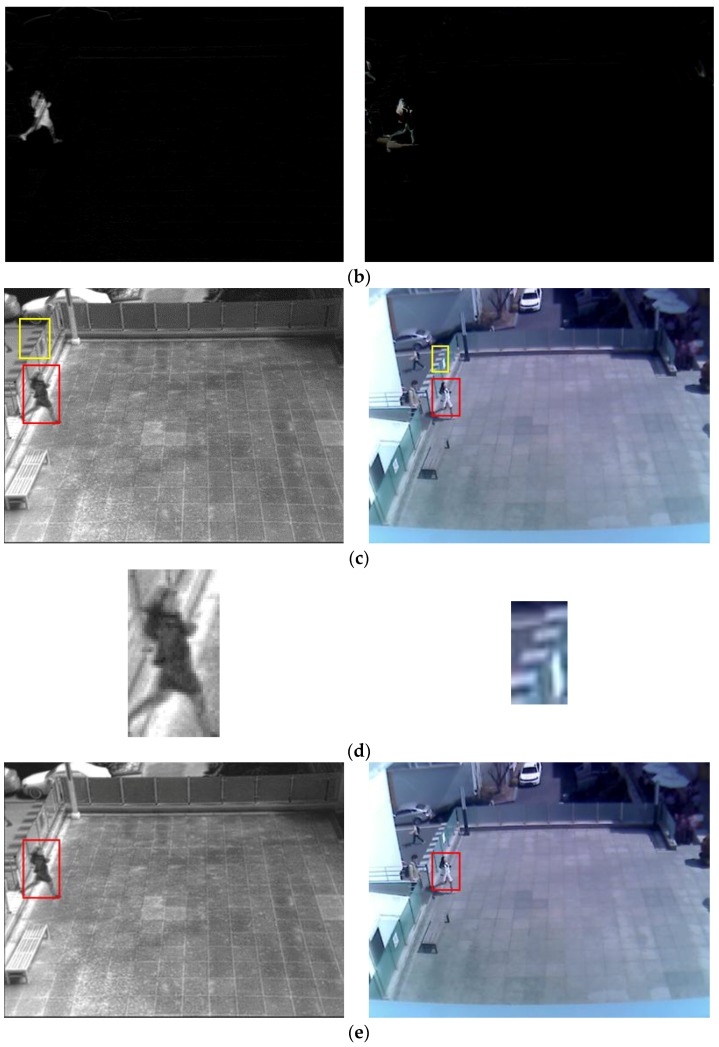
Images to illustrate the steps shown in Figure 1: (**a**) Two input images, (**b**) Two difference images, (**c**) Detected candidate boxes by background subtraction and noise reduction. (**d**) Selected candidate boxes by FIS, which are used as CNN inputs. (**e**) Final detected area of containing the pedestrian by CNN.

Figure 1 describes the overall procedure of the proposed system. The system receives the data from both visible light and FIR light images through dual cameras (step (1) and Figure 2a). It detects the candidate based on background subtraction and noise reduction by using difference images (Figure 2b) between the background image and the input images [12]. Here, the mean value of the candidate within the difference image obtained from the visible light image is “feature 1”, and that gained by the FIR light image is “feature 2”. In general, the mean value of the difference images increases along with the increase of difference between the pedestrian and the background, which causes the consequent increment of possibility of correct pedestrian. However, as shown in Figure 2c, the output candidate exists not only in the red box (pedestrian candidate) but also in the yellow box (non-pedestrian candidate).

As mentioned earlier, the pedestrian candidate usually has a high mean value in the difference image while the non-pedestrian candidate has a low mean value in the difference image as shown in Figure 2b. Nevertheless, because all the regions inside pedestrian candidate do not show high mean value in the difference image of Figure 2b, a low threshold value for image binarization should be used to correctly detect the whole regions inside pedestrian candidate, which causes the incorrect detection of non-pedestrian candidate as pedestrian one as shown in Figure 2c. It is difficult to correctly discriminate between the pedestrian and non-pedestrian candidates, and the FIS is designed using the mean value of the gradient magnitude of pedestrian or non-pedestrian candidate in difference images as “feature 3”. The system adaptively selects a more appropriate candidate to be verified by the CNN between the two boxes of Figure 2c—after adding “feature 3” as weights, and using the FIS with “feature 1” and “feature 2” as an input (see step (3) of Figure 1). Then, it uses the selected candidates of pedestrian and non-pedestrian (Figure 2d) as the pre-trained input for the CNN to ultimately classify it into a pedestrian or non-pedestrian case (see step (4) of Figure 1 and Figure 2e).

### 3.2. Adaptive Selection by FIS

The FIS in this paper is designed to adaptively select one candidate between two pedestrian candidates derived from visible light and FIR camera images, which is deemed most appropriate for the pedestrian detection process. Table 2 presents a fuzzy rule table designed through this research to be used for the FIS. This research uses two features, and has “Low” and “High” as inputs, and “Low” “Medium” and “High” as outputs. The two features consist of “feature 1”, a mean value of the candidate gained from the visible light image, and “feature 2”, a mean value from the FIR light image. That is because, in general, the bigger the difference between the pedestrian and the background is, the bigger the mean value in difference image is, meaning that the outcome is more likely to be the correct pedestrian. 

For instance, as listed in Table 2a, when “feature 1” and “feature 2” are “Low” (a lower mean value) and “High” (a higher mean value), respectively, the difference between the pedestrian and the background of the FIR light images is larger than that of the visible light image. Therefore, the output value becomes “High” meaning that the candidate of the FIR light image is selected. However, the opposite case implies that the difference of the visible light image is larger than that of the FIR light image. The output value becomes “Low” which in other words implies that the candidate of the visible light image is selected. If the “feature 1” and “feature 2” are both “Low” or “High”, it is difficult to determine which candidate is more desirable (between the two candidates of visible light and FIR light images), giving the output a “Medium” Value. 

However, as shown in Figure 2c, the selected candidate is present not only in the pedestrian candidate (the red box) but also in the non-pedestrian candidate (the yellow box). Although the pedestrian candidate has high mean value in the difference image as mentioned before, the non-pedestrian candidate has a low mean value as shown in Figure 2b. Considering that, this study designs the rule table for non-pedestrian features as shown in Table 2b in order to have opposite features from Table 2a.

In general, when the FIS uses two inputs, it employs the IF-THEN rule [28], and the output will be produced by AND or OR calculation depending on the relationship between the FIS inputs. This research selected an AND calculation among the IF-THEN rules as the FIS makes adaptive selection while considering “feature 1” and “feature 2” together.

**Figure 3:**
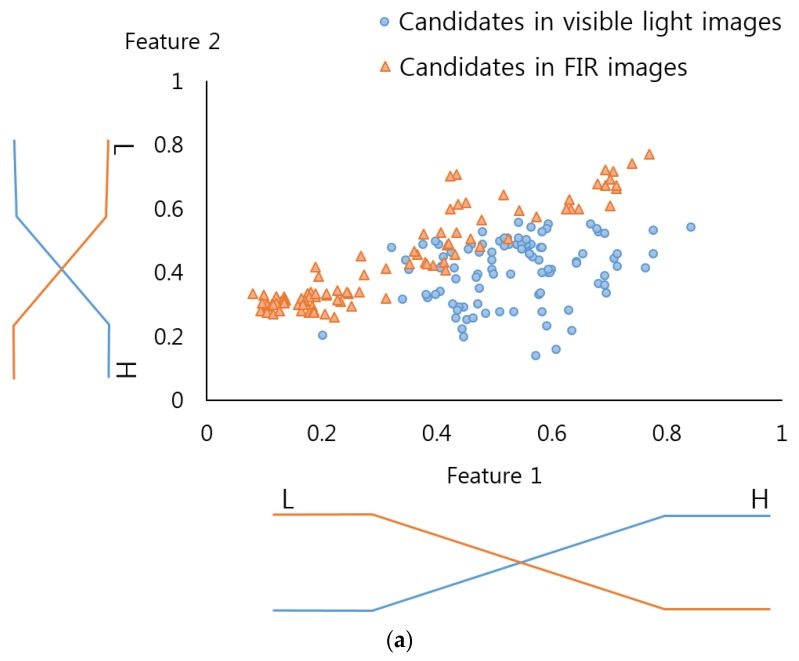

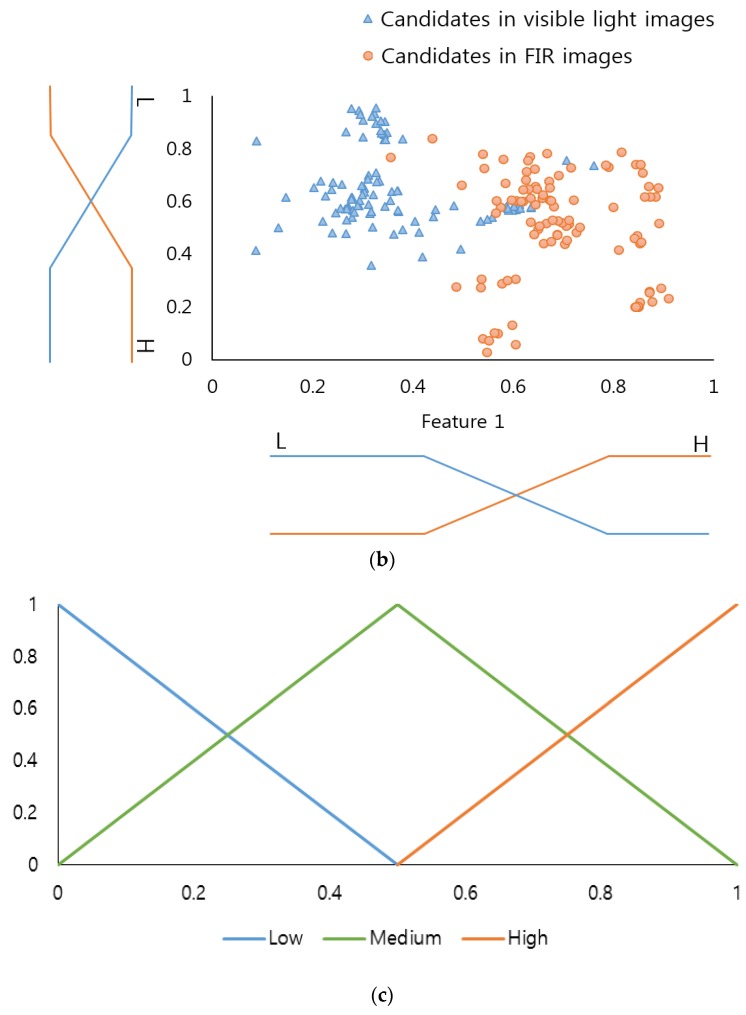
Membership functions. Input membership function (**a**) for pedestrians; (**b**) for non-pedestrian features. (**c**) Output membership function.

Figure 3 describes the linear membership function used in this research, which is widely used in the FIS as its calculation speed is very fast and its algorithm is less complex compared to the non-linear membership function [29,30,31]. As mentioned, the input images have pedestrian and non-pedestrian categories, and two fuzzy rule tables (see Table 2) were designed to reflect the differences in their features. In this regard, two input membership functions were used: one for the pedestrian and the other for the non-pedestrian. In order to more accurately determine the frame of the linear input membership function, this study gained a data distribution for “feature 1” and “feature 2” (see Figure 3a,b) by using part of the training data of the CNN(to be illustrated in Section 3.3). Based on this, each linear input membership function for pedestrian and non-pedestrian is separately (“Low”, “High”) designed. Also, as shown in Figure 3c, the output membership functions were designed for three outputs, “Low” “Medium” and “High”. Figure 3c is not related to the data of Figure 3a,b. In conventional fuzzy inference system, the output membership function is usually designed heuristically. Therefore, we use the three linear membership functions, which have been widely used in the fuzzy inference system.

The “feature 1” (*f1*) and “feature 2” (*f2*) in this research can be “Low” and “High” each shown in Table 2. Therefore, their outputs become (G*_f1_*^L^(*f1*), G*_f1_*^H^(*f1*)) and (G*_f2_*^L^(*f2*), G*_f2_*^H^(*f2*)) due to function (G*_f1_*^L^(⋅),G*_f1_*^H^(⋅),G*_f2_*^L^(⋅), and G*_f2_*^H^(⋅)) of the input membership of Figure 3a,b. Four pairs of combinations were obtained from this and these became (G*_f1_*^L^(*f1*), G*_f2_*^L^(*f2*)), (G*_f1_*^L^(*f1*), G*_f2_*^H^(*f2*)), (G*_f1_*^H^(*f1*), G*_f2_*^L^(*f2*)), and (G*_f1_*^H^(*f1*), G*_f2_*^H^(*f2*)). The fuzzy rule table of the Max and Min rules [29], and the Table 2 help gain four inference values from four pairs of combinations.

**Figure 4:**
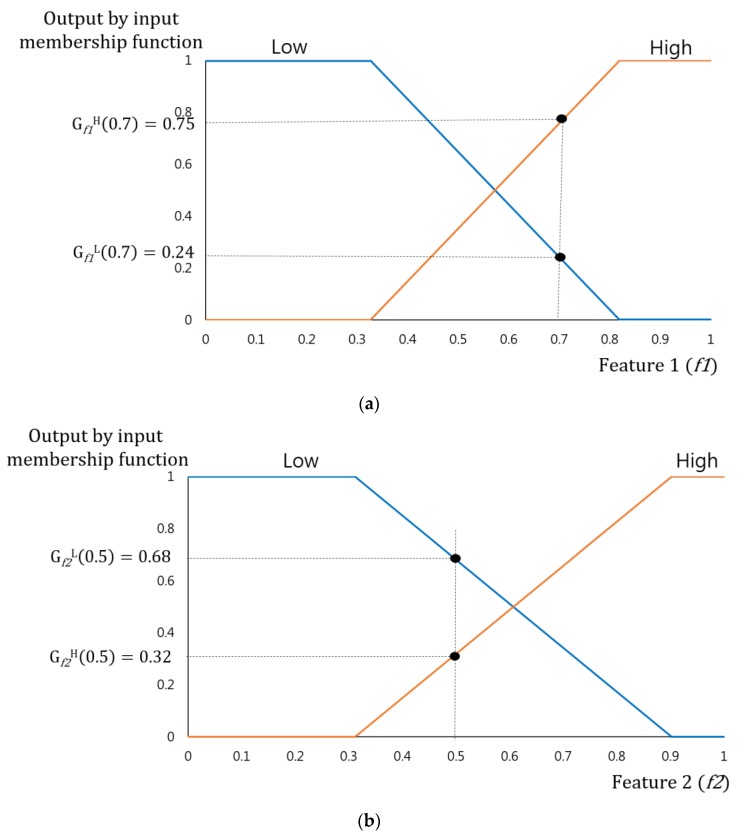
Example of obtaining outputs by input membership functions. (**a**) Output of “Feature 1”. (**b**) Output of “Feature 2”.

For instance, when *f1* = 0.7, *f2* = 0.5 as shown in Figure 4, the output value gained by the input membership function becomes (G*_f1_*^L^(0.7) = 0.24, G*_f1_*^H^(0.7) = 0.75), (G*_f2_*^L^(0.5) = 0.68, G*_f2_*^H^(0.5) = 0.32). As mentioned earlier, these four output values lead to four combinations, including (0.24(L), 0.68(L)), (0.24(L), 0.32(H)), (0.75(H), 0.68(L)), (0.75(H), 0.32(H)). An inference value may be determined for each combination according to Min rule, Max rule, and fuzzy rule table of Table 2. If (0.24(L), 0.68(L)), when applying the Min rule and the fuzzy rule of Table 2b (IF “Low” and “Low”, THEN “Medium”), inference value will be determined as 0.2 (M). If (0.75(H), 0.68(L)) and applying the Max rule and the fuzzy rule of Table 2a (IF “High” and “Low”, THEN “Low”), the inference value will be 0.75(L). Likewise, the inference value resulting from the four combinations are described in Table 3 and Table 4. 

**Figure 5:**
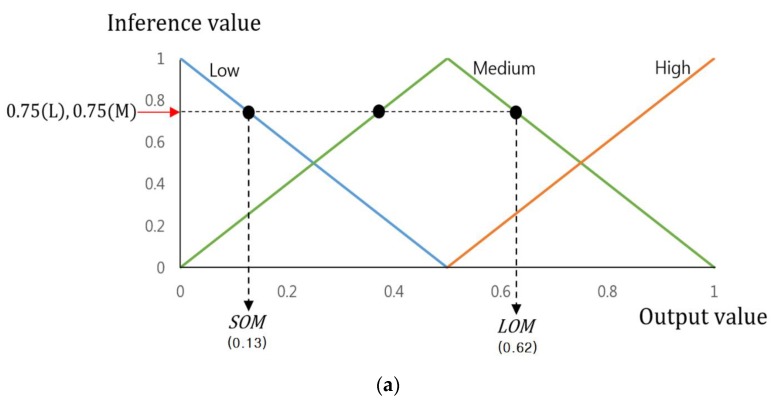

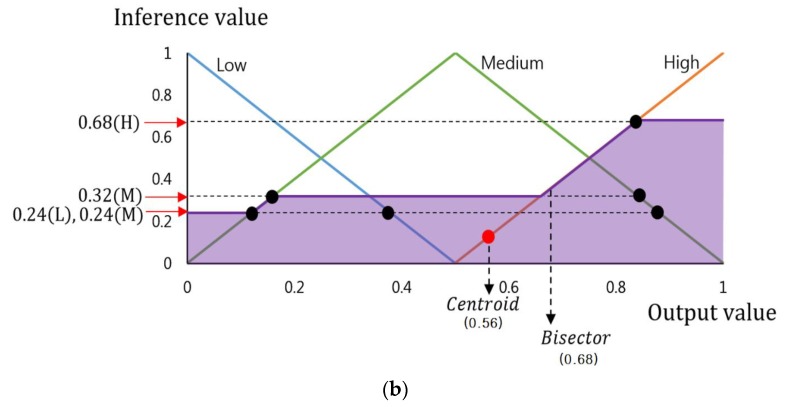
An example of Output Value depending on various Defuzzification methods (**a**) Output values by SOM and LOM methods with the inference values by Max rule of Table 3. (**a**) Output values by Centroid and Bisector methods with the inference values by Min rule of Table 4.

Therefore, the final output value of the FIS will be calculated through various defuzzification and the output membership function with its input of the inference values as shown in Figure 5. This study employed the smallest of maximum (SOM), the middle of maximum (MOM), the largest of maximum (LOM), Bisector, and Centroid methods, most widely used among various defuzzification methods [32,33,34]. Among those, the SOM, MOM, and LOM methods establish the FIS output values by maximum inference values among many inference values. The SOM and LOM methods establish the final output values using the smallest and largest values, which are gained by maximum inference. The MOM method uses the average value of the smallest and largest as the final output value. Figure 5a is an example of a defuzzification process based on the inference values by Max rule of Table 3 (0.32(H), 0.68(M), 0.75(L), and 0.75(M)). This figure only uses these values as its maximum inference values are 0.75(L) and 0.75(M). Therefore, as shown in Figure 5a, two output values (0.13 and 0.62) are produced by SOM and LOM methods, and their average value is gained as (0.375 = (0.13 + 0.62)/2) by MOM method.

Bisector and centroid methods are the means to determine the FIS output value by using all the inference values. The centroid method determines the FIS output value based on the geometric center of the area from the area (the purple colored area of Figure 5a) defined by all the inference values. The bisector method identifies the FIS output value based on the line dividing the defined area into two having the same size. Figure 5b is an example of a defuzzification process based on the inference values by Min rule of Table 4 (0.24(L), 0.24(M), 0.32(M), and 0.68(H)), which produces two output value (0.56 and 0.68) by the centroid and bisector methods. 

As seen in Figure 2c, the produced candidate area exists not only in the red box (pedestrian candidate) but also in the yellow box (non-pedestrian candidate). As mentioned earlier, the pedestrian candidate has a higher mean value in the difference image while the non-pedestrian candidate has a low mean value just as Figure 2b. In the current level, it is possible to know whether the produced candidate area is under a pedestrian or a non-pedestrian category. Therefore, in order to design the FIS based on that, this study used the mean value of the gradient magnitude in the difference image within the produced candidate as “feature 3”. By reflecting such a “feature 3” as a weight into the FIS output value, as shown in Figure 5, this work makes an adaptive selection among the two candidates, (the yellow and red boxes of Figure 2c), which results in one appropriate candidate for verification by the CNN. 

**Figure 6:**
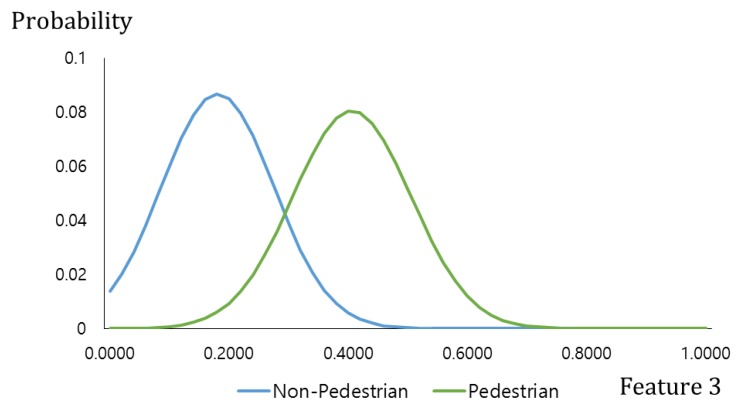
Data distribution pf feature 3.

Figure 6 describes two distributions of “feature 3”, produced from the pedestrian and the non-pedestrian data used in Figure 3a,b by using a Gaussian fitting. Similar to the difference image of Figure 2b, the gradient magnitude of the pedestrian candidate is generally higher than that of the non-pedestrian candidate. Therefore, the pedestrian distribution is on the right side of the non-pedestrian distribution as shown in Figure 6. 

In this study, the FIS output value for the pedestrian, shown in Figure 5, is defined as $o_{p}$ and the FIS output value for the non-pedestrian is defined as $o_{n-p}$. It defines the probability for finding a pedestrian to be (via Figure 6), and the probability for finding a non-pedestrian as $p_{p}$ and $p_{n-p}$, respectively. This leads to the final output value ($o_{\mathrm{FIS}}$) given through Equation (1):

(1) $$o_{\mathrm{FIS}}=\;\frac{o_{p}\times p_{p}+o_{n-p}\times p_{n-p}}{p_{p}+p_{n-p}}$$

Finally, as given in Equation (2), the system adaptively selects one candidate that is more appropriate for the CNN-based classification of pedestrian and non-pedestrian. This selection is done between two (pedestrian) candidates in visible light and FIR images. The optimal threshold of Equation (2) is experimentally determined based on the pedestrian and non-pedestrian data used in Figure 3a,b:

(2) $$\mathrm{Selected candidate}=\{\begin{array}{c} \mathrm{Candidate in visible light image, if}\;o_{\mathrm{FIS}}<\mathrm{Threshold}\\ \mathrm{Candidate in FIR image, otherwise} \end{array}$$

### 3.3. Classification of Pedestrian and Non-Pedestrian by CNN

**Figure 7:**
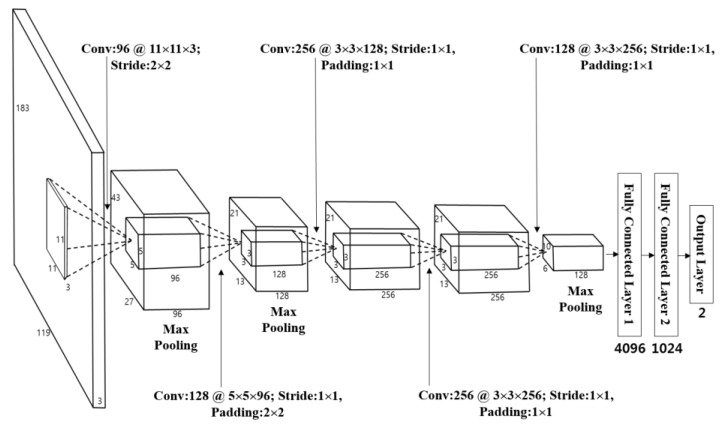
The CNN architecture.

This research uses a CNN in order to classify the chosen candidate by Equation (2). The classification yields whether the candidate is of pedestrian or non-pedestrian (background) category. As shown in Figure 2d, the candidate can be gained by visible light image or the FIR image. Therefore, the candidate from the visible light image is used as the CNN input learned through the visible light image training set. On the other hand, the candidate from the FIR image is used as the input learned through the FIR image training set. Both structures are equal and are illustrated in Table 5 and Figure 7. 

As seen in this table and figure, the CNN in this research includes five convolutional layers and three fully connected layers [35]. Input images are the pedestrian and the non-pedestrian candidate images. As each input candidate image has a different size, this paper considers the ratio of the width and length of the general pedestrian, and resizes them into 119 pixels (width), 183 pixels (height), three (channels) through bilinear interpolation.

Several previous studies, including AlexNet [36] and others [37,38], used a square shape with the same width and length as input images. However, the general pedestrian area, which this study aims to find, has longer length than its width. Therefore, when normalizing the size into a square shape, the image is unacceptably stretched toward its width compared to its length, and distorts its pedestrian area, making it difficult to extract the features accurately. Also, when selecting the CNN input image as a square shape without stretching toward the width direction, the background area (especially, on the left and right to the pedestrian), is heavily reflected on the output yielding inaccurate features. Considering this aspect, this study uses the pedestrian or the non-pedestrian images with a normalized size of 119-by-183 pixels (width-by-height) as the CNN input. Through such size normalization, when the object’s size changes depending on its location relative to the camera, such change can be compensated. In addition, this study normalized the brightness of the input image by the zero-center method discussed in [39]. The 119-by-183 pixels (width-by-height) used in this method is much smaller than the 227-by-227 pixels (height-by-width) discussed in AlexNet [36]. Therefore, we can significantly reduce the number of filters in each convolution layers and the number of nodes in fully-connected layers than those in stated in the AlexNet. Also, AlexNet was designed in order to classify 1000 classes. However, this research can reduce the training time as it can distinguish only two classes of the pedestrian and non-pedestrian areas [35].

In the 1st convolutional layer, 96 filters with the size of 11 × 11 × 3 are used at a stride of 2 × 2 pixels in the horizontal and vertical directions. The size of the feature map is 55 × 87 × 96 in the 1st convolutional layer, such that 55 and 87 are the output width and height, respectively. The calculations are based on: (output width (or height) = (input width (or height) − filter width (or height) + 2× padding)/stride + 1 [40]). For instance, in Table 5, input height, filter height, padding, and stride are 183, 11, 0, and 2, respectively. Therefore, the output height becomes 87 ((183 − 11 + 2× 0)/2 + 1). Unlike the previous studies [41,42], this research relatively enlarges the filter size of the 1st convolutional layer as the input image is very dark with high level of noise by its nature. Therefore, the enlarged filter can control the feature, which can be extracted wrongly due to the noise. Therefore, a rectified linear unit (ReLU) layer is used for the calculation as given by Equation (3) [43,44,45]:

(3) y = max(0,x)

where x and y are the input and output values, respectively. This formula can lessen the vanishing gradient problem [46], which can cause a faster processing speed than a non-linear activation function [35].

The local response normalization layer is used behind the ReLU layer, as described in Table 5, which has a formula as follows:

(4) $$b_{x,y}^{i}=\frac{a_{x,y}^{i}}{{\left(p+\alpha \sum_{j=max\left(0,\;i-\frac{n}{2}\right)}^{min\left(N-1,\;i+\frac{n}{2}\right)}{\left(a_{x,y}^{j}\right)}^{2}\right)}^{\beta }}$$

In Equation (4), $b_{x,y}^{i}$ is a value obtained by normalization [36]. In this research, we used 1, 0.0001, and 0.75 for the values of *p*, α, and β, respectively. $a_{x,y}^{i}$ is the neuron activity computed by the application of the *i*th kernel at the location (x, y), and it performs normalization for the adjacent *n* kernel maps at the identical spatial position [36]. In this study, *n* was set as 5. N implies the total number of filters in the layer. In order to make the CNN structure resilient to the image translation and local noise, the feature map gained through the local response normalization layer goes through the max pooling layer as given in Table 5. Max pooling layer uses the output after selecting the maximum value among the figures within the defined mask ranges. This is similar to conducting a subsampling. Once it goes through the Max pooling layer, it will produce 96 feature maps with sizes of 27 × 43 pixels as shown in Table 5 and Figure 7. 

In order to fine-tune the 1st convolutional layer, as given in Table 5 and Figure 7, the 2nd convolutional layer that has 128 filters with a size of 5 × 5 × 96, a stride of 1 × 1 pixels (in the horizontal and vertical directions), and a padding of 2 × 2 pixels (in the horizontal and vertical directions) can be used behind the 1st convolutional layer. Similar to the 1st convolutional layer, after going through ReLU, cross channel normalization, and max pooling layers, we obtained 128 feature maps with the size of 13 × 21 pixels as shown in Figure 7 and Table 5. The first two layers are used to extract the low-level image features, such as blobs texture feature or edges.

Then, three additional convolutional layers are used for the high-level feature extraction as given in Figure 7 and Table 5. In details, the 3rd convolutional layer adopts 256 filters with the size of 3 × 3 × 128, the 4th convolutional layer has 256 filters with the size of 3 × 3 × 256, and the 5th convolutional layer uses 128 filters with the size of 3 × 3 × 256. 

Through these five convolutional layers, 128 feature maps with the size of 6 × 10 pixels are finally obtained, which are fed to the additional three fully connected layers including 4096, 1024, and 2 neurons, respectively. This research will finally classify two classes of pedestrian areas and non-pedestrian areas through a CNN. Therefore, the last (3rd) fully connected layer (called as “output layer”) of Figure 7 and Table 5 has only two nodes. The 3rd fully connected layer uses Softmax function, as given through Equation (5) [44]:

(5) $$\sigma {\left(s\right)}_{j}=\frac{e^{sj}}{\sum_{n=1}^{K}e^{sn}}$$

Given that the array of the output neurons is set as s, we can obtain the probability of neurons belonging to the jth class by dividing the value of the jth element by the summation of the values of all the elements. As illustrated in the previous studies [36,47], the CNN-based recognition system has an over-fitting problem, which can cause low recognition accuracy with testing data although the accuracy with the training data is still high. To address such problems, this research employs data augmentation and dropout methods [36,47], which can reduce the effects of over-fitting problem. More details about the outcome of the data augmentation are presented in Section 4.1. For the dropout method, we adopt the dropout probability of 50% to disconnect the connections several neurons between previous layer and the next layers in the fully connected network [35,36,47]. 

## 4. Experimental Result

### 4.1. Experimental Data and Training

**Figure 8:**
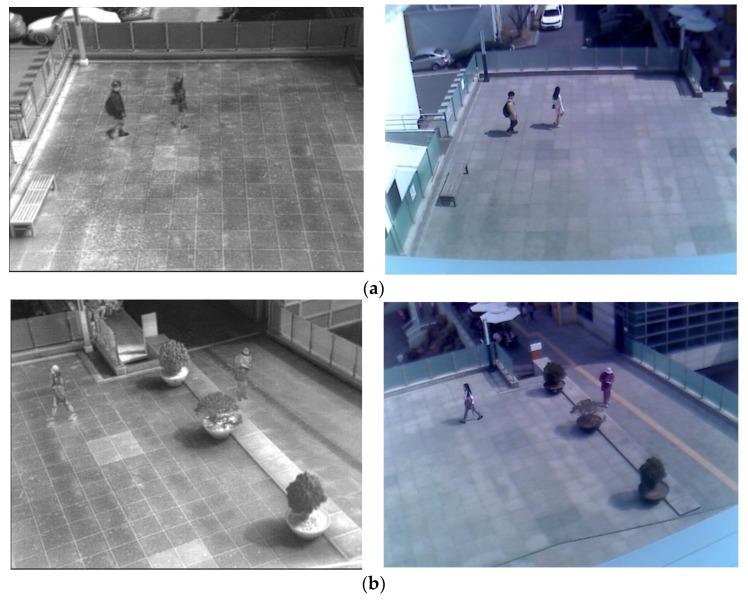

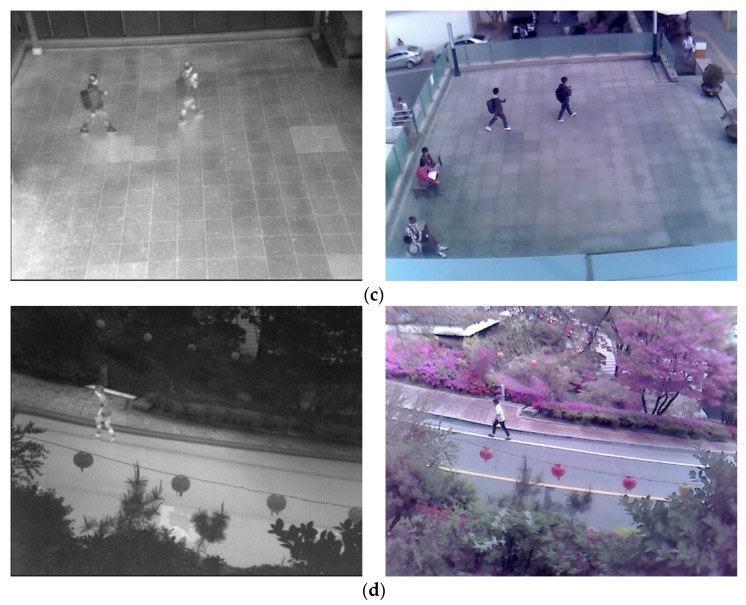
Example of various images in the experimental DVLFPD-DB1 used in this study: (**a**) Sub-database 1, (**b**) Sub-database 2, (**c**) Sub-database 3, and (**d**) Sub-database 4.

Table 6 and Figure 8 show the sample images from the database (DVLFPD-DB1), which were used in this study. This database is built independently by our lab, and is available with our trained CNN model to other researchers through [48] for the purposes of comparisons by other researchers. In total, there are four sub-databases, and the total number of frames of visible light images and FIR images is 4080 each. 

To obtain the images, this study used a dual camera system [12] consisting of a Tau640 FIR camera (19 mm, FLIR, Wilsonville, OR, USA) [49], and a C600 visible light web-camera (Logitech, Lausanne, Switzerland) [50]. In order to record the filming conditions, a WH-1091 wireless weather station (Chanju Tech., Paju-si, Gyeonggi-do, Korea) was used [51].

This research conducted the CNN training, and the tests in such a way that a four-fold cross validation can be achieved by using the four sub-databases as shown in Figure 8. In addition, it conducted a data augmentation step in order to solve the overfitting issue when conducting the CNN training. For data augmentation, image translation and cropping was used based on previous research [36]. In other words, the study gained four additional augmented candidate images from a single original candidate image listed in Table 5. This was achieved by adjusting five pixel translations and cropping to box locations (up, down, right and left) that contained the pedestrian and the non-pedestrian candidates. The augmented data were used only for the CNN training. For testing purposes, non-augmented original candidate images were used. 

The experimental conditions in this research were as follows: all the tests were conducted in a desktop computer consisting of Intel^®^ Core™ i7-3770K CPU @ 3.50 GHz (four CPUs), main memory of 16 GB, and a GeForce GTX 1070 (1,920 CUDA cores) graphics card (NVIDIA, Santa Clara, CA, USA) with memory of 8 GB [52]. The algorithms of the CNN training and testing were implemented by Window Caffe (version 1) [53].

This study used stochastic gradient descent (SGD) method for the CNN training [54]. The SGD method is a tool to find the optimal weight, which minimizes the difference between the desired and the calculated outputs based on the derivatives [35]. 

**Figure 9:**
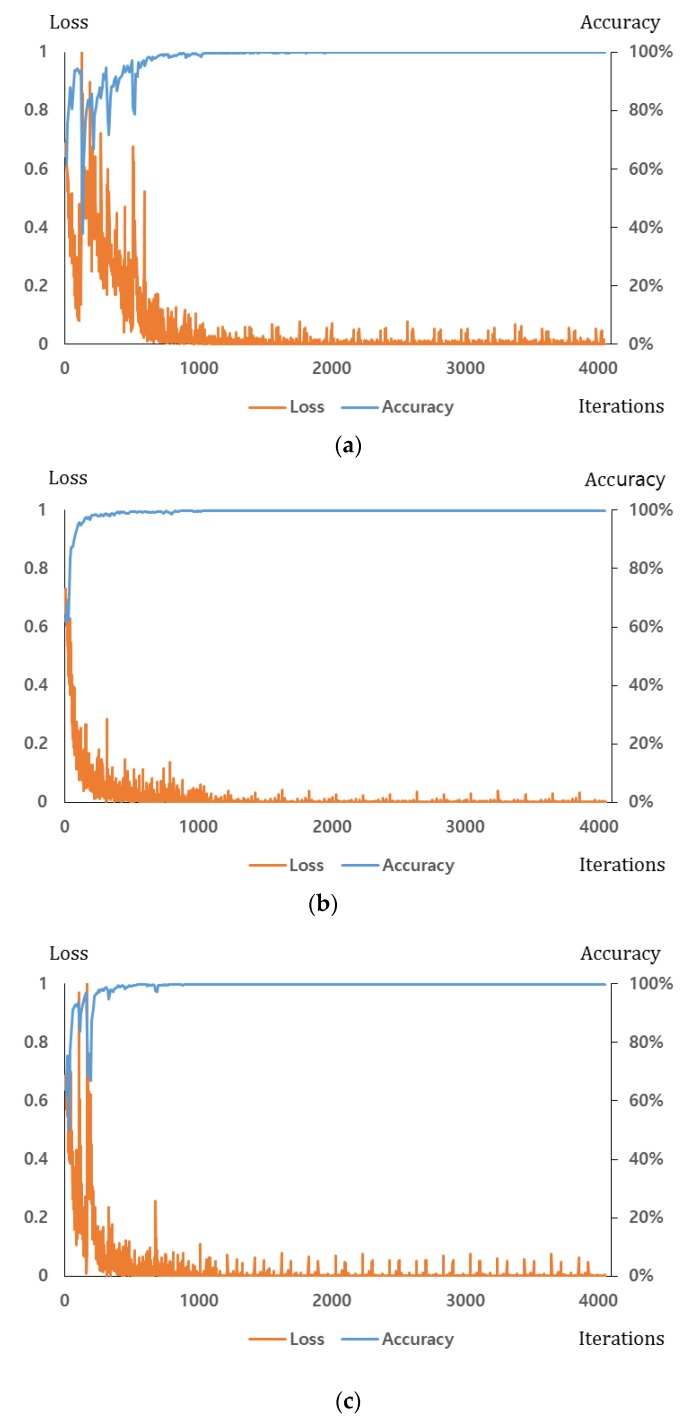

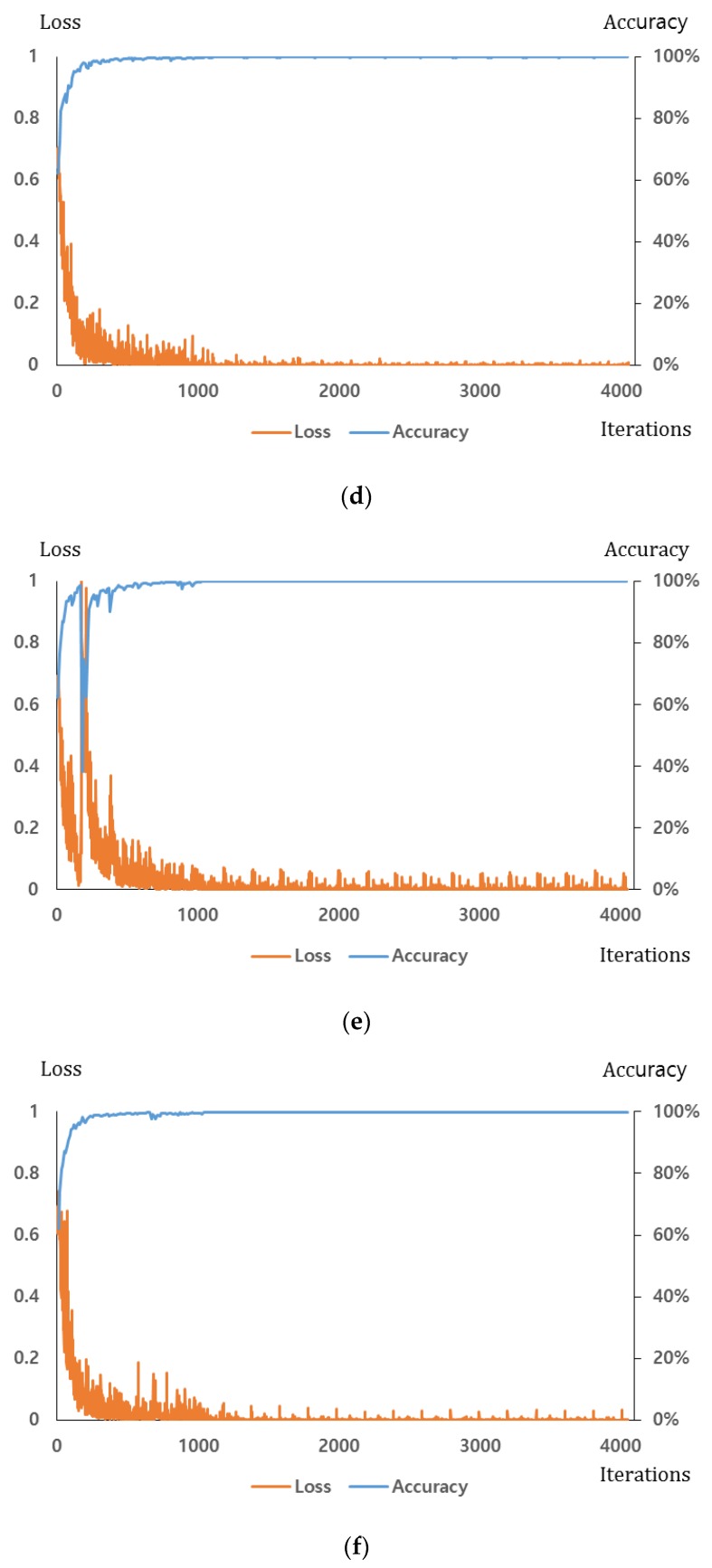

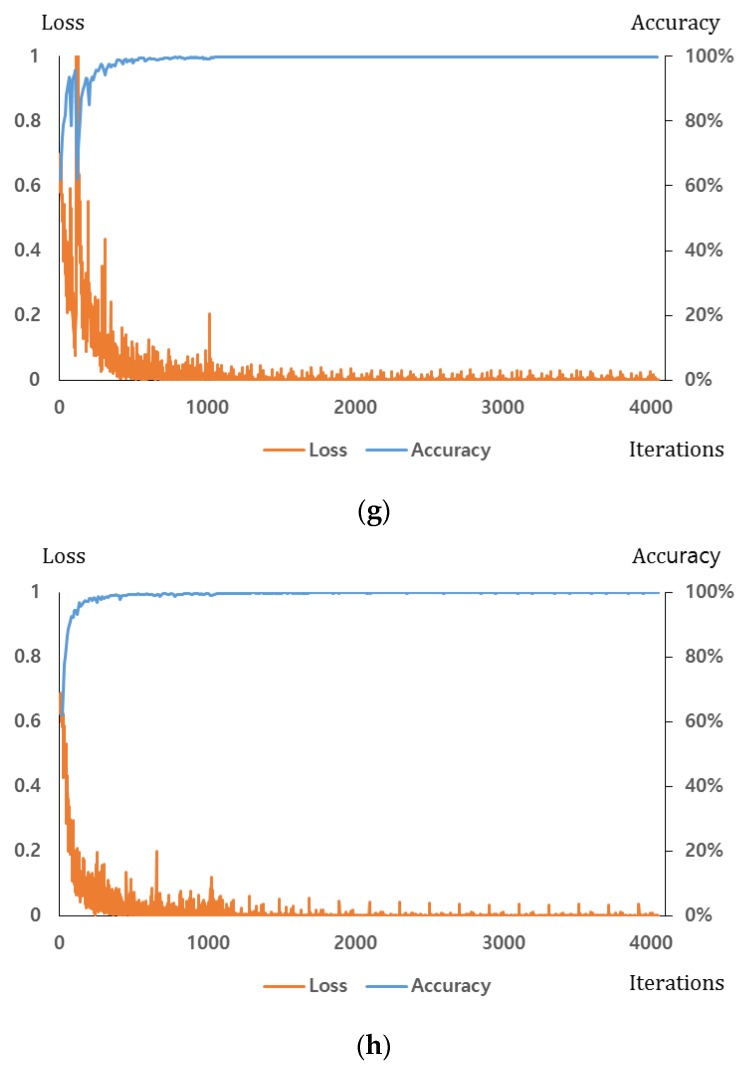
Loss and accuracy graphs of the training procedure: (**a**) the 1st fold (visible light candidate images); (**b**) the 1st fold (FIR candidate image); (**c**) the 2nd fold (visible light candidate images); (**d**) the 2nd fold (FIR candidate image); (**e**) the 3rd fold (visible light candidate images); (**f**) the 3rd fold (FIR candidate image); (**g**) the 4th fold (visible light candidate images); (**h**) the 4th fold (FIR candidate image).

Unlike the gradient descent (GD) method, the SGD method defines the total number of iterations by dividing the training set by the mini-batch size, sets the training completion time until it reaches the total number of iterations (set as 1 epoch), and conducts the training for the preset number of epoch. The CNN training parameters are as follows: base_lr = 0.01, lr_policy = step, minibatchsize = 128, stepsize = 1013 (5 epoch), max_iter = 4054 (20 epoch), momentum = 0.9, gamma = 0.1, weight_decay = 0.0001, regularization_type = L2. The detail explanations of these parameters can be found in the following literature [53]. Figure 9 shows the loss and the training accuracy for the CNN training process along with the number of iterations. The loss graph converges toward 0, and the training accuracy reaches 100% as the iteration of the four folds increase. At this condition, the CNN is considered to be fully trained. 

**Figure 10:**
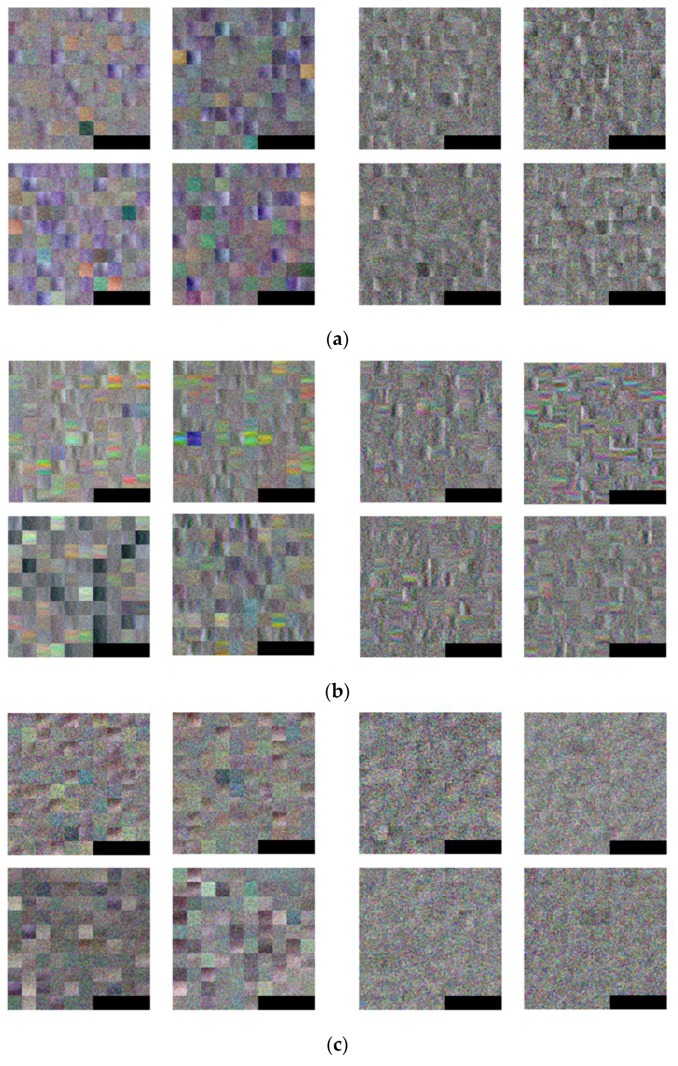
Examples of 96 filters obtained from the 1st convolution layer through training with (**a**) original DVLFPD-DB1, (**b**) degraded DVLFPD-DB1, and (**c**) OSU color-thermal database. In (**a**–**c**), left four images show the 96 filters obtained by training with visible light candidate images whereas right 4 images represent those by training with FIR candidate images. In the left and right four images, the left-upper, right-upper, left-lower, and right-lower images show the 96 filters obtained by training of 1st~4th fold cross validation, respectively.

Figure 10 shows an example of 96 filters with 11 × 11 × 3 (as shown in Table 4) in the 1st convolutional layer, as identified through the training. For the purposes of visibility, the filters are resized five times as larger by bi-linear interpolation. In this study, the experiments used three types of databases, (a) the original DVLFPD-DB1, (b) the degraded DVLFPD-DB1 (see Section 4.2), which reflects Gaussian noise and Gaussian blurring into the original database, and (c) the open database (see Section 4.2), or the Ohio State University (OSU) color-thermal database [55]. Therefore, Figure 10 presents 96 filters - each gained from the CNN training by using these three types of databases. As shown in the following Table 7 of Section 4.1, the Bisector method has the highest performance among those various defuzzification methods, and therefore, Figure 10 shows the shape of filter when using the Bisector method. By comparing the Figure 10a,b, the shapes of filters eligible for edge detection in Figure 10a is more distinctive than those in Figure 10b. That is because the edge strength in the degraded DVLFPD-DB1 is reduced by image blurring compared to that in the original DVLFPD-DB1. 

**Figure 11:**
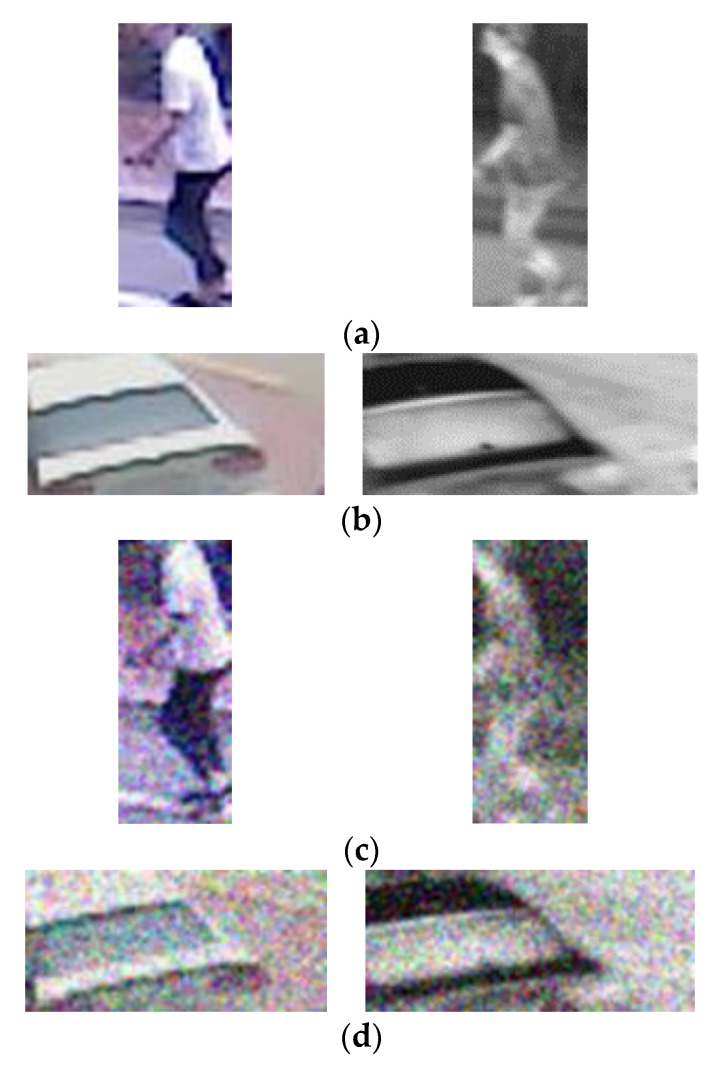
Examples of original and degraded DVLFPD-DB1. Original images of (**a**) pedestrian candidate, and (**b**) non-pedestrian candidate. Degraded images of (**c**) pedestrian candidate, and (**d**) non-pedestrian candidate. In (**a**–**d**), left and right images show the candidates from visible light and FIR light images, respectively.

**Figure 12:**
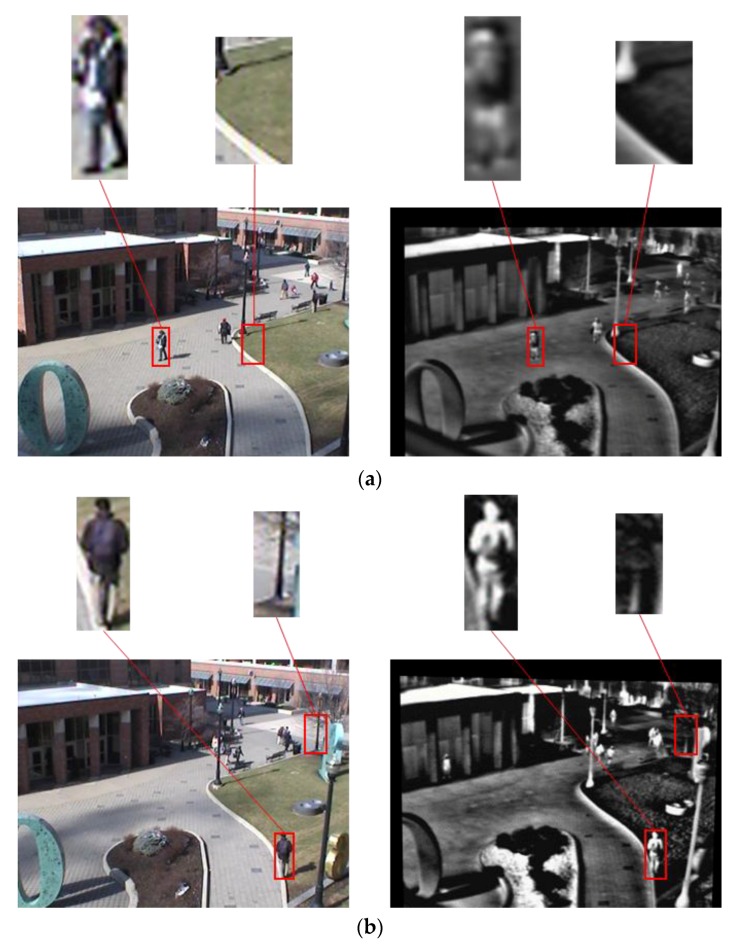

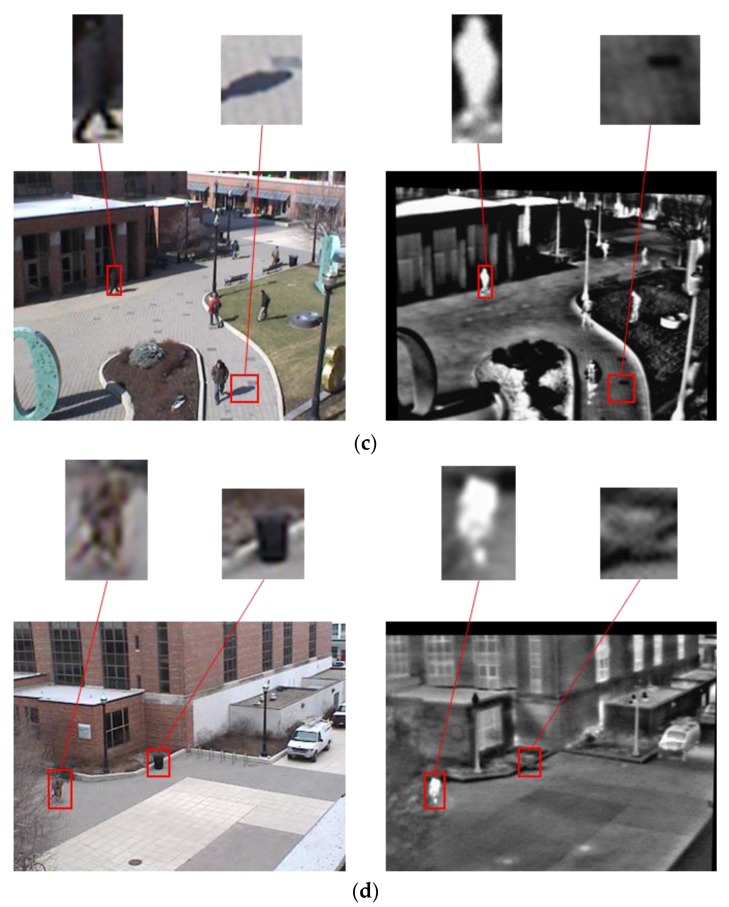
Examples of OSU color-thermal database. (**a**) Example 1, (**b**) example 2, (**c**) example 3, and (**d**) example 4. In (**a**–**d**), left and right images show the visible light and FIR light images, respectively. In (**a**–**d**), upper and lower images represent the candidates and original images, respectively.

In addition, by comparing the shapes of filters of Figure 10a–c, we can find that the shapes of left four filters of Figure 10c from OSU color-thermal database is simpler than those of Figure 10a,b. In addition, the shapes of right four filters of Figure 10c do not show the characteristics of direction compared to those of Figure 10a,b. That is because the pedestrian or non-pedestrian candidates in OSU color-thermal database is smaller than those in the original DVLFPD-DB1 and the degraded DVLFPD-DB1 as shown in Figure 8, Figure 11 and Figure 12. Therefore, more local features are extracted from OSU color-thermal database through CNN training to discriminate the pedestrian and non-pedestrian candidates than those from the original DVLFPD-DB1 and the degraded DVLFPD-DB1.

### 4.2. Testing of the Proposed Method

The classification accuracy from the FIS’s defuzzification method, proposed as the first test, is measured and presented in Table 7. This study defines the pedestrian and the non-pedestrian candidates as positive and negative data in order to test their performances. They are also defined as true negative (TN), true positives (TP), false negatives (FN), and false positives (FP). TN is the case where the background (non-pedestrian) candidate is correctly recognized as the background region, whereas TP is the case where the pedestrian candidate is correctly recognized as the pedestrian region. FN is the case where the pedestrian candidate is incorrectly recognized as the background region, whereas FP is the case where the background (non-pedestrian) candidate is incorrectly recognized as the pedestrian region. Based on these, we can define two errors of false negative rate (FNR) and false positive rate (FPR). In addition, two accuracies of true positive rate (TPR) and true negative rate (TNR) can be defined. In other words, TPR and TNR are calculated as 100-FNR (%) and 100-FPR (%) respectively.

Table 7 shows TPR, TNR, FNR, and FPR after processing through the confusion matrix. For instance, according to the LOM method in Table 7, TPR, TNR, FNR, and FPR are 99.74%, 99.35%, 0.26%, and 0.65%, respectively. Table 7 presents the average value of the four testing accuracies produced by the four-fold cross validation. The test showed that the bisector method has a higher classification accuracy compared to the other methods. Based on this, this study evaluated the testing performance by using the bisector method-based FIS.

The second test compared the classification accuracies among the HOG-SVM-based method [18,22], the CNN and single camera-based method (visible light or FIR camera) [6,10], and the late fusion CNN-based method [13], which are widely used in the previously reported pedestrian detection studies. For fair comparisons, the same augmented data (as reported in the previous studies [6,10,13,18,22]) were used in our method. In addition, the same testing data were used for our method and the previous methods. Table 8 shows the average value of the four testing accuracies produced by the four-fold cross validation. As described in Table 8, the proposed method is far more accurate than the previously studied methods. 

Also, for performance comparisons, this research used precision, recall, accuracy, and F1 score as given in Table 9. With TP, TN, FP, and FN, we have used the following four criteria for accuracy measurements [56]:

(6) $$\mathrm{Precision}=\frac{\#\mathrm{TP}}{\#\mathrm{TP}+\#\mathrm{FP}}$$

(7) $$\mathrm{Recall}=\frac{\#\mathrm{TP}}{\#\mathrm{TP}+\#\mathrm{FN}}$$

(8) $$\mathrm{Accuracy}\;\left(\mathrm{ACC}\right)=\frac{\#\mathrm{TP}+\#\mathrm{TN}}{\#\mathrm{TP}+\#\mathrm{TN}+\#\mathrm{FP}+\#\mathrm{FN}}$$

(9) $$F1\;\mathrm{score}=2\cdot \frac{\mathrm{Precision}\cdot \mathrm{Recall}}{\mathrm{Precision}+\mathrm{Recall}}$$

where #TP, #TN, #FP, and #FN mean the numbers of TP, TN, FP, and FN, respectively. Minimum and maximum values of precision, recall, accuracy, and F1 score are 0 (%) and 100 (%), respectively, where 0 (%) and 100 (%) represent the lowest and highest accuracies, respectively. Table 9 shows the average value of the four testing accuracies produced by the four-fold cross validation. As described in Table 9, the proposed method is significantly more accurate than the previous methods. 

As the third experiment, this research created the degraded dataset artificially including Gaussian noise (sigma of 0.03) and Gaussian blurring (sigma of 0.5) in order to account for more environmental variables into the original dataset and evaluate them for their accuracy. Such factors have negative effects as they are able to exist in the actual intelligent surveillance camera system environment. Therefore, in order to exhibit a strong performance under such a poor condition, this study created a degraded dataset as shown in Figure 11. 

Table 10 and Table 11 show the average value of the four testing accuracies gained by the four-fold cross validation. As showed in Table 10 and Table 11, even in the case of using the degraded dataset, the proposed method had better classification accuracy than the other methods. 

The fourth experiment is based on the open database (OSU color-thermal database) [55] such that a fair comparison can be done by other researchers. As shown in Figure 12, OSU color-thermal database is an image gained by the FIR camera and visible light camera in the fixed outdoor with various environmental factors.

Table 12 and Table 13 show the average value of the four testing accuracies gained by the four-fold cross validation. As Table 12 and Table 13 present, the proposed method shows a higher accuracy even with the OSU color-thermal database.

**Figure 13:**
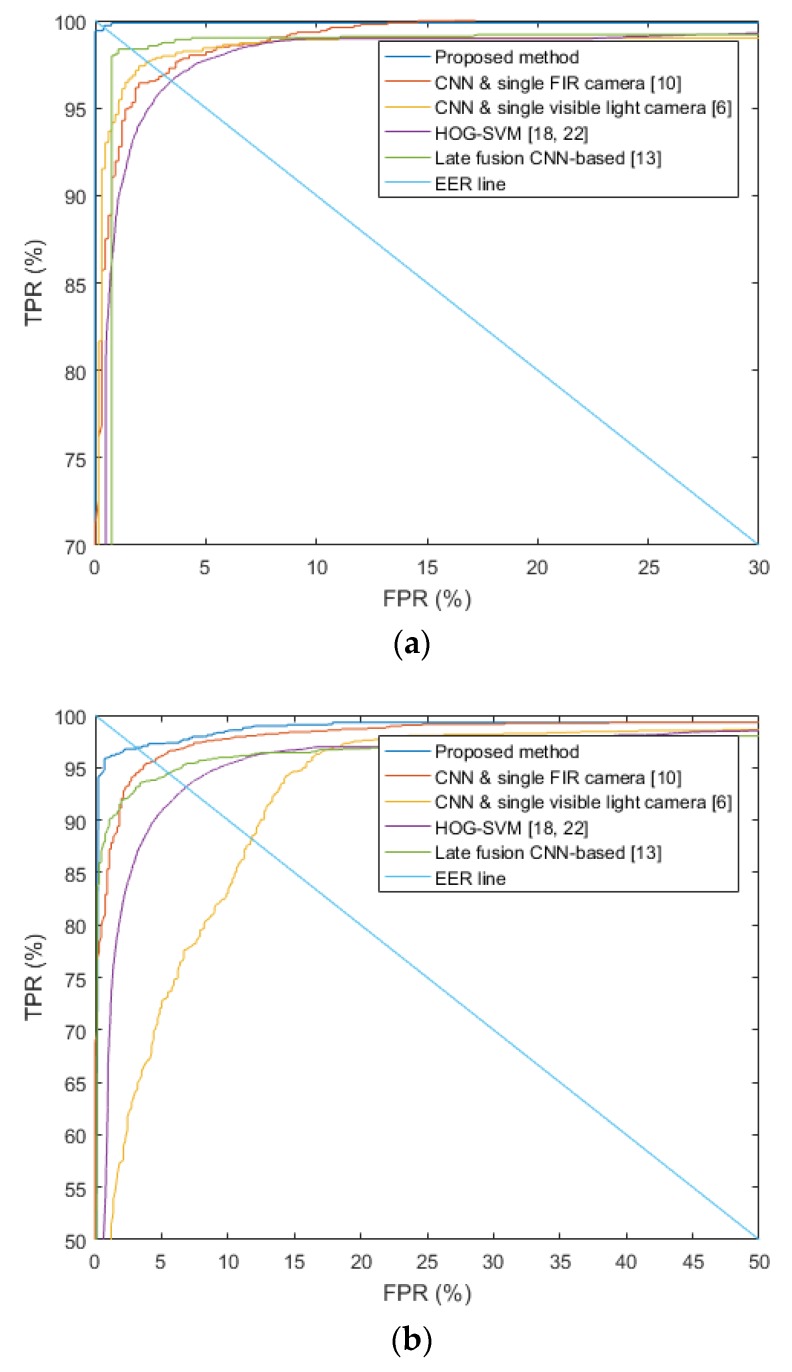

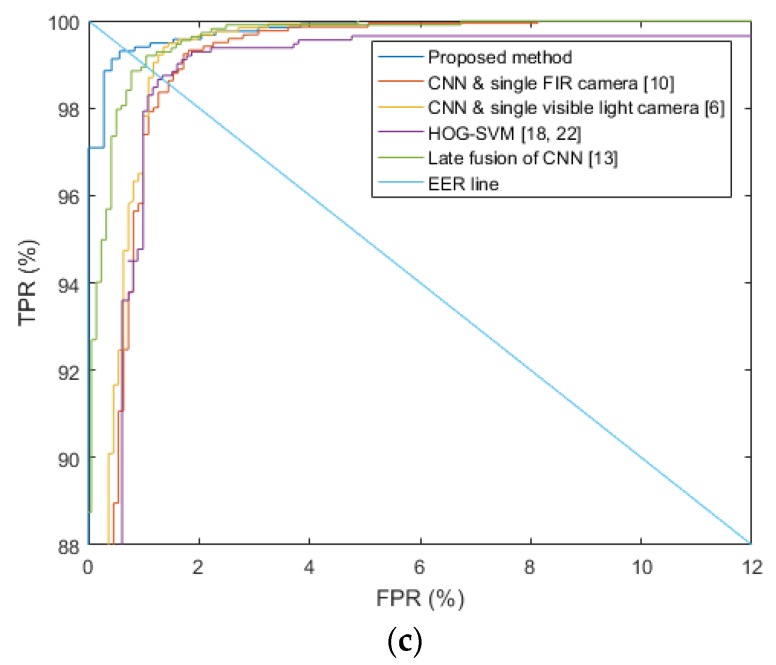
ROC curves with (**a**) original DVLFPD-DB1, (**b**) degraded DVLFPD-DB1, and (**c**) OSU color-thermal database.

Figure 13 shows TPR and FPR-based receiver operation characteristic (ROC) curves among the proposed method and the others with regard to three types of the databases. The figure presents the average graph of the four testing accuracies gained by the four-fold cross validation. 

As explained before, FNR (100-TPR (%)) has the trade-off relationship with FPR. According to threshold of classification, larger FNR causes smaller FPR, and vice versa. Equal error rate (EER) is the error rate (FNR or FPR) when FNR is same to FPR. As shown in Figure 13, the accuracy of the proposed method is significantly higher than that of the previous methods. 

**Figure 14:**
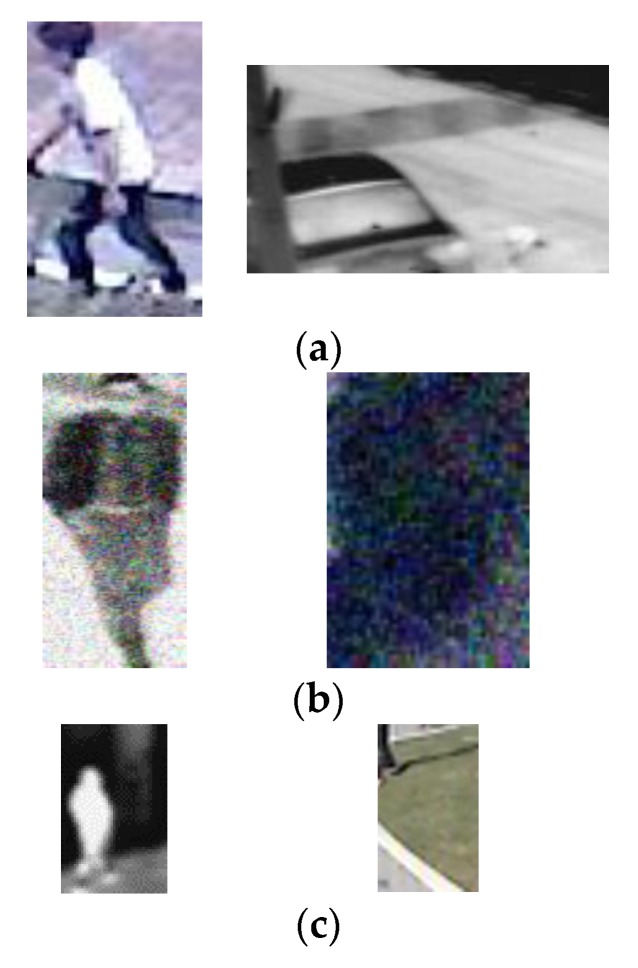
Examples of correct classification with (**a**) original DVLFPD-DB1, (**b**) degraded DVLFPD-DB1, and (**c**) OSU color-thermal database. In (**a**–**c**), left and right images show the examples of TP and TN candidates, respectively.

Figure 14 shows the examples of correct classification. Although the candidates were obtained in various environments of noise, blurring, size, and illuminations, all the cases of TP and TN are correctly recognized.

**Figure 15:**
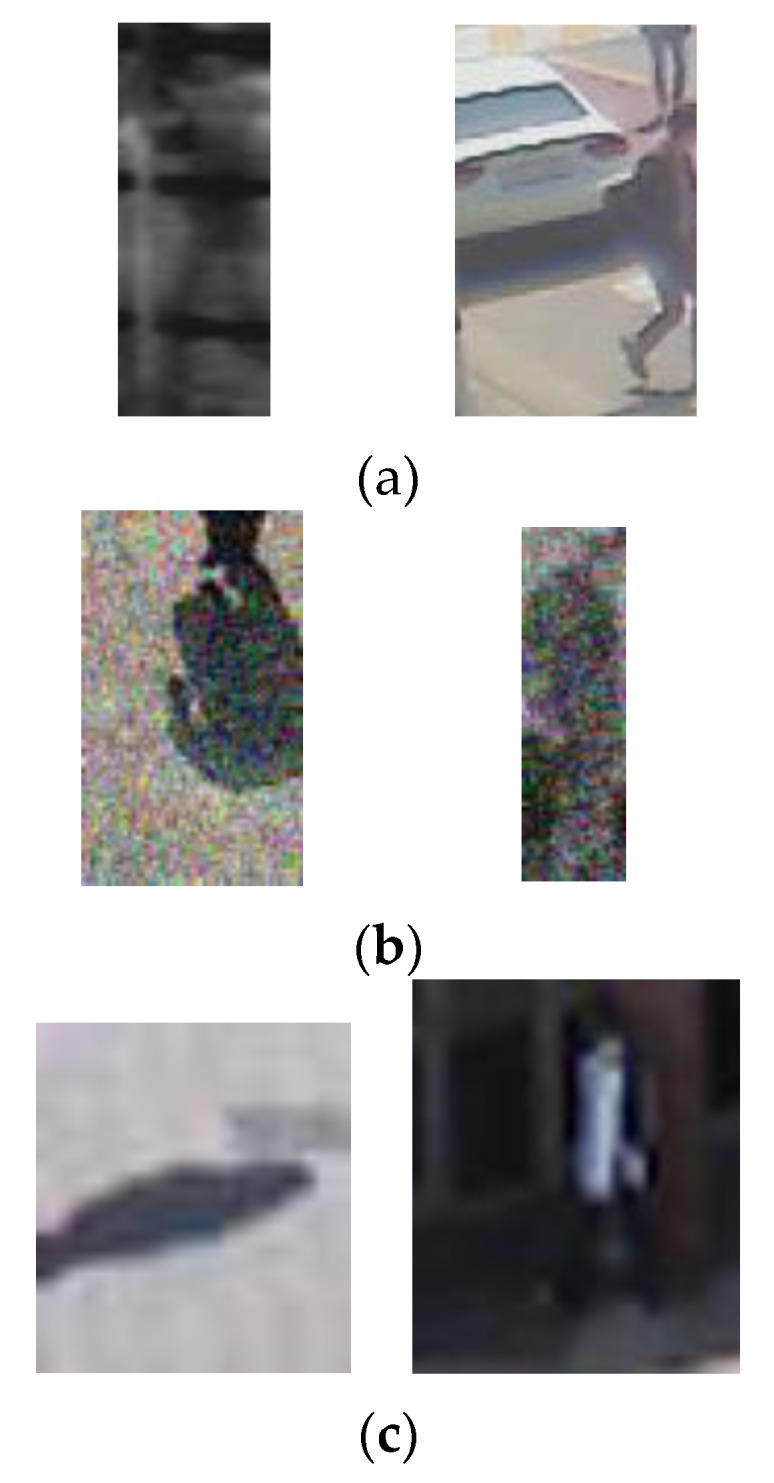
Examples of incorrect classification with (**a**) original DVLFPD-DB1, (**b**) degraded DVLFPD-DB1, and (**c**) OSU color-thermal database. In (**a**–**c**), left and right images show the FP and FN cases, respectively.

Figure 15 shows the examples of incorrect classification. In Figure 15a–c, the left and right images show the FP and FN cases, respectively. The FP errors happen when the shape of background is similar to a pedestrian (Figure 15a), lots of noise are included (Figure 15b), and the shape of a shadow is similar to that of a pedestrian (Figure 15c). The FN errors occur when the part of pedestrian is occluded in the candidate box (Figure 15a), lots of noises are included (Figure 15b), and a large background area is included in the detected pedestrian box (Figure 15a,c). 

## 5. Conclusions

This paper made an adaptive selection to find the most appropriate candidate for pedestrian detection among the two pedestrian candidates of visible light and FIR camera images by using the FIS and suggested a new method to verify that candidate with the CNN. In order to test the accuracy of the algorithm under the various conditions, the study used not only the independently designed DVLFPD-DB1 but also the degraded DVLFPD-DB1 combining the original DVLFPD-DB1 with Gaussian blurring and noise. Also, the OSU color-thermal database, an open database, was used as well in order to compare the accuracy of the proposed method with the others. 

CNN has been widely used for its performance in various fields. However, intensive training is required for the usage of CNN with lots of training data. In many applications, it is often the case that collecting lots of training data is a difficult procedure, so a subsequent data augmentation process is performed. To lessen this disadvantage of CNN-based methods, we have made our trained CNN model with our collected DVLFPD-DB1 and degraded one by Gaussian blurring and noise publically available to other researchers for the purpose of performing comparisons. In future work, the proposed method can form the basis for studying crime recognition and face detection of criminals. Further, there are plans to conduct research to sense emergency situations in vehicular environments by detecting various subjects through the front camera in the vehicle in order to utilize the proposed method for a driver assistance system. 

## Figures and Tables

**Table 1:** Comparisons of the proposed and the previously researched methods.

Category	Methods		Advantage	Disadvantage
Single camera-based	AdaBoost cascade [17]		- Faster processing speeds. - Better performance under low image resolutions.	- Affected by various environmental changes, such as, changing lighting and shadows, and cases where the background temperature is similar to that of the pedestrians’ body.
	HOG–SVM [18,22], integral HOG [19], neural network based on receptive fields [20], and background generation [21]		- More resilient in simple conditions. - Faster processing speed than multiple camera-based algorithm.	
	CNN-based method [6,10]		More accurate than the past single camera-based method.	
Multiple camera-based	Stereo visible light cameras	Shape and texture information [23]	Better detect pedestrians as it is able to utilize more information than the single camera-based method.	- Longer time to process as it has to process both the camera images.
	Visible light & NIR cameras	HOG-SVM [22]		
	Visible light & FIR cameras	Tetra-vision-based HOG-SVM [24]	Better night vision pedestrian detection inside the car.	- No performance without vehicle headlight. - High number of calculation is required as it needs to process two camera images.
		Camera selection [11]	Better performance under various conditions.	- Detection capability is affected as it has no final verification process for the detected pedestrian area.
		Difference image-based fusion [12]		
		Late fusion CNN-based method [13]	Higher CNN-based detection accuracy.	- Processing hours and algorithm complexity increases as the method processes input from two camera images to conduct CNN twice.
		Proposed method	- Increased detection reliability (compared to the single camera-based method) by means of adaptively selecting one candidate between two pedestrian candidates received from visible light and FIR camera images. Applies a FIS, and reduces algorithm complexity and processing time. - More resilient detection capability under various environmental changes by means of intensively training and using a diverse dataset.	Design of the fuzzy rule tables and membership function is needed for the FIS.

**Table:** (**a**)

**Input**		**Output**
**Feature 1**	**Feature 2**	
Low	Low	Medium
Low	High	High
High	Low	Low
High	High	Medium

**Table:** (**b**)

**Input**		**Output**
**Feature 1**	**Feature 2**	
Low	Low	Medium
Low	High	Low
High	Low	High
High	High	Medium

**Table 3:** An example of the Inference Value produced by Min and Max rules with fuzzy rule table of Table 2a.

Feature 1	Feature 2	Inference Value	
		Min Rule	Max Rule
0.24(L)	0.68(L)	0.24(M)	0.68(M)
0.24(L)	0.32(H)	0.24(H)	0.32(H)
0.75(H)	0.68(L)	0.68(L)	0.75(L)
0.75(H)	0.32(H)	0.32(M)	0.75(M)

**Table 4:** An example of the Inference Value produced by Min and Max rules with fuzzy rule table of Table 2b.

Feature 1	Feature 2	Inference Value	
		Min Rule	Max Rule
0.24(L)	0.68(L)	0.24(M)	0.68(M)
0.24(L)	0.32(H)	0.24(L)	0.32(L)
0.75(H)	0.68(L)	0.68(H)	0.75(H)
0.75(H)	0.32(H)	0.32(M)	0.75(M)

**Table 5:** CNN architecture.

Layer Type	Number of Filters	Size of Feature Map (Width × Height × Channel)	Size of Filter	Stride	Padding
Image input layer		119 × 183 × 3			
1st convolutional layer	96	55 × 87 × 96	11 × 11 × 3	2 × 2	0 × 0
Rectified linear unit (ReLU) layer		55 × 87 × 96			
Local response normalization layer		55 × 87 × 96			
Max pooling layer	1	27 × 43 × 96	3 × 3	2 × 2	0 × 0
2nd convolutional layer	128	27 × 43 × 128	5 × 5 × 96	1 × 1	2 × 2
ReLU layer		27 × 43 × 128			
Local response normalization layer		27 × 43 × 128			
Max pooling layer	1	13 × 21 × 128	3 × 3	2 × 2	0 × 0
3rd convolutional layer	256	13 × 21 × 256	3 × 3 × 128	1 × 1	1 × 1
ReLU layer		13 × 21 × 256			
4th convolutional layer	256	13 × 21 × 256	3 × 3 × 256	1 × 1	1 × 1
ReLU layer		13 × 21 × 256			
5th convolutional layer	128	13 × 21 × 128	3 × 3 × 256	1 × 1	1 × 1
ReLU layer		13 × 21 × 128			
Max pooling layer	1	6 × 10 × 128	3 × 3	2 × 2	0 × 0
1st fully connected layer		4096			
ReLU layer		4096			
2nd fully connected layer		1024			
ReLU layer		1024			
Dropout layer		1024			
3rd fully connected layer		2			
Softmax layer		2			
Classification layer (output layer)		2			

**Table 6:** Description of the database.

		Sub-Database 1	Sub-Database 2	Sub-Database 3	Sub-Database 4
Number of image		598	651	2364	467
Number of pedestrian candidate		1123	566	2432	169
Number of non-pedestrian candidate		763	734	784	347
(range of width) × (range of height) (pixels)	Pedestrian	(27 ~ 91) × (87 ~ 231)	(47 ~ 85) × (85 ~ 163)	(31 ~ 105) × (79 ~ 245)	(30 ~ 40) × (90 ~ 120)
	Non-pedestrian	(51 ~ 161) × (63 ~ 142)	(29 ~ 93) × (49 ~ 143)	(53 ~ 83) × (55 ~ 147)	(60 ~ 170) × (50 ~ 110)
Weather Conditions		Surface temperature: 30.4 °C, Air temperature: 22.5 °C, Wind speed: 10 km/h, Sensory temperature: 21.3 °C	Surface temperature: 25.5 °C, Air temperature: 24 °C, Wind speed: 5 km/h, Sensory temperature: 23.5 °C	Surface temperature: 20 °C, Air temperature: 21 °C, Wind Speed: 6.1 km/h, Sensory temperature: 21 °C	Surface temperature: 16 °C, Air temperature: 20.5 °C, Wind Speed: 2.5 km/h, Sensory temperature: 20.8 °C

**Table 7:** Classification accuracies for defuzzification method (unit: %).

Defuzzification Method			Recognized		Avg. of TPR and TNR
			Pedestrian	Non-Pedestrian	
Actual	LOM	Pedestrian	99.74	0.26	99.55
		Non-pedestrian	0.65	99.35	
	MOM	Pedestrian	99.74	0.26	99.55
		Non-pedestrian	0.65	99.35	
	SOM	Pedestrian	99.74	0.26	99.57
		Non-pedestrian	0.61	99.39	
	Centroid	Pedestrian	99.72	0.28	99.58
		Non-pedestrian	0.57	99.43	
	Bisector	Pedestrian	99.63	0.37	99.61
		Non-pedestrian	0.41	99.59	

**Table 8:** Comparisons of classification accuracies with original DVLFPD-DB1 based on confusion matrix (unit: %).

Method			Recognized		Avg. of TPR and TNR
			Pedestrian	Non-Pedestrian	
Actual	HOG-SVM based [18,22]	Pedestrian	97.86	2.14	96.28
		Non-pedestrian	5.31	94.69	
	CNN and single visible light camera-based [6]	Pedestrian	97.69	2.31	97.59
		Non-pedestrian	2.52	97.48	
	CNN and single FIR camera-based [10]	Pedestrian	96.53	3.47	96.95
		Non-pedestrian	2.64	97.36	
	Late fusion CNN-based [13]	Pedestrian	98.38	1.62	98.03
		Non-pedestrian	2.33	97.67	
	Proposed method	Pedestrian	99.63	0.37	99.61
		Non-pedestrian	0.41	99.59	

**Table 9:** Comparisons of classification accuracies with original DVLFPD-DB1 based on precision, recall, accuracy, and F1 score (unit: %).

Method	Precision	Recall	ACC	F1 Score
HOG-SVM based [18,22]	96.78	97.86	96.66	97.32
CNN and single visible light camera-based [6]	98.42	97.69	97.58	98.05
CNN and single FIR camera-based [10]	98.32	96.53	96.37	97.42
Late fusion CNN-based [13]	98.57	98.38	98.11	98.47
Proposed method	99.75	99.63	99.61	99.69

**Table 10:** Comparisons of classification accuracies with degraded DVLFPD-DB1 based on confusion matrix (unit: %).

Method			Recognized		Avg. of TPR and TNR
			Pedestrian	Non-Pedestrian	
Actual	HOG-SVM based [18,22]	Pedestrian	96.11	3.89	92.13
		Non-pedestrian	11.85	88.15	
	CNN and single visible light camera-based [6]	Pedestrian	97.32	2.68	89.16
		Non-pedestrian	19.01	80.99	
	CNN and single FIR camera-based [10]	Pedestrian	96.62	3.38	95.33
		Non-pedestrian	5.97	94.03	
	Late fusion CNN-based [13]	Pedestrian	95.96	4.04	93.01
		Non-pedestrian	9.94	90.06	
	Proposed method	Pedestrian	96.33	3.67	97.23
		Non-pedestrian	1.88	98.12	

**Table 11:** Comparisons of classification accuracies with degraded DVLFPD-DB1 based on precision, recall, accuracy, and F1 score (unit: %).

Method	Precision	Recall	ACC	F1 Score
HOG-SVM based [18,22]	92.98	96.11	93.09	94.52
CNN and single visible light camera-based [6]	89.29	97.32	91.10	93.13
CNN and single FIR camera-based [10]	96.31	96.62	95.63	96.46
Late fusion CNN-based [13]	94.03	95.96	93.72	94.99
Proposed method	98.80	96.33	97.02	97.55

**Table 12:** Comparisons of classification accuracies with OSU color-thermal database based on the confusion matrix (unit: %).

Method			Recognized		Avg. of TPR and TNR
			Pedestrian	Non-Pedestrian	
Actual	HOG-SVM based [18,22]	Pedestrian	99.11	0.89	98.69
		Non-pedestrian	1.73	98.27	
	CNN and single visible light camera-based [6]	Pedestrian	99.64	0.36	98.75
		Non-pedestrian	2.14	97.86	
	CNN and single FIR camera-based [10]	Pedestrian	99.51	0.49	98.54
		Non-pedestrian	2.44	97.56	
	Late fusion CNN-based [13]	Pedestrian	99.28	0.72	98.90
		Non-pedestrian	1.49	98.51	
	Proposed method	Pedestrian	99.58	0.42	99.02
		Non-pedestrian	1.54	98.46	

**Table 13:** Comparisons of classification accuracies with OSU color-thermal database based on precision, recall, accuracy, and F1 score (unit: %).

Method	Precision	Recall	ACC	F1 Score
HOG-SVM based [18,22]	98.34	99.36	98.83	98.85
CNN and single visible light camera-based [6]	97.86	99.64	98.74	98.74
CNN and single FIR camera-based [10]	97.62	99.51	98.54	98.56
Late fusion CNN-based [13]	98.53	99.28	98.83	98.90
Proposed method	98.52	99.58	99.03	99.05
